# Robotic ankle control can provide appropriate assistance throughout the gait cycle in healthy adults

**DOI:** 10.3389/fnbot.2022.993939

**Published:** 2022-09-27

**Authors:** Kei Nakagawa, Keita Higashi, Akari Ikeda, Naoto Kadono, Eiichiro Tanaka, Louis Yuge

**Affiliations:** ^1^Division of Bio-Environmental Adaptation Sciences, Graduate School of Biomedical and Health Sciences, Hiroshima University, Hiroshima, Japan; ^2^Department of Rehabilitation, Innoshima Medical Association Hospital, Onomichi, Japan; ^3^Graduate School of Information, Production and Systems, Faculty of Science and Engineering, Waseda University, Kita-Kyushu, Japan

**Keywords:** gait, ankle joint, walking assistive device, rocker function, kinematic analysis

## Abstract

Ankle foot orthoses are mainly applied to provide stability in the stance phase and adequate foot clearance in the swing phase; however, they do not sufficiently assist during the entire gait cycle. On the other hand, robotic-controlled orthoses can provide mechanical assistance throughout the phases of the gait cycle. This study investigated the effect of ankle control throughout the gait cycle using an ankle joint walking assistive device under five different robotic assistance conditions: uncontrolled, dorsiflexion, and plantar flexion controlled at high and low speeds in the initial loading phase. Compared with the no-control condition, the plantar flexion condition enhanced knee extension and delayed the timing of ankle dorsiflexion in the stance phase; however, the opposite effect occurred under the dorsiflexion condition. Significant differences in the trailing limb angle and minimum toe clearance were also observed, although the same assistance was applied from the mid-stance phase to the initial swing phase. Ankle assistance in the initial loading phase affected the knee extension and ankle dorsiflexion angle during the stance phase. The smooth weight shift obtained might have a positive effect on lifting the limb during the swing phase. Robotic ankle control may provide appropriate assistance throughout the gait cycle according to individual gait ability.

## Introduction

Smooth walking requires proper movement of the ankle joint. The rocker function plays an important role as a rotation axis for shock absorption in the early stance and in generating an anterior propulsive force in the late stance and pre-swing phases (Czerniecki, 1988).

Post-stroke hemiplegic patients often face difficulties in walking and utilize characteristic walking strategies due to sensory dysfunction, muscle weakness, and spasticity of the ankle dorsiflexor and plantar flexor muscles. They often lack the typical heel strike and push-off movements that play important roles in heel and forefoot rocker function (Wong et al., 2004). From the viewpoint of knee movement, walking strategy can be divided into three patterns: stiff knee pattern (SKP), extension thrust pattern (ETP), and buckling knee pattern (BKP) (De Quervain et al., 1996). SKP is characterized by insufficient knee flexion during the swing phase, which is caused by overactivity of the knee extensors and inadequate push-off function in the late stance phase due to spasticity of the plantar flexor muscles (Kerrigan et al., 1991; Caty et al., 2008; Gatti et al., 2012; Campanini et al., 2013). ETP is characterized by a stronger hip extensor moment than the knee extensor moment and prolonged biceps femoris activity. Insufficient activity of the ankle joint induced by plantar flexion contracture with or without spasticity and weakness of the dorsiflexion muscles are considered to be major causes of poor control of the tibia (Mulroy et al., 2003; Kinsella and Moran, 2008). BKP with knee hyperflexion in the mid-stance phase is caused by weakness of the hip and knee extensors, which prolongs the activity of the quadriceps muscles to support flexed knee posture. Weakness of the plantar flexor muscles, which reduces the poor plantarflexion angle during the loading response, is considered to be one of the causes (Mulroy et al., 2003; Kinsella and Moran, 2008). Therefore, controlling the movement of the ankle joint is important to compensate for walking in patients with hemiplegia.

The use of ankle foot orthoses (AFO) tuned for individual walking disabilities compensates for gait disturbance by adequately controlling the motion of the tibia during the stance phase (Mulroy et al., 2010; Nolan and Yarossi, 2011; Alam et al., 2014; Daryabor et al., 2018). For instance, AFOs with plantar flexion resistance (AFO-PR) with an oil damper or spring are considered to assist in the insufficient eccentric contraction of the dorsiflexors in the loading response phase (Yokoyama et al., 2005; Yamamoto et al., 2009, 2015; Ohata et al., 2011; Kobayashi et al., 2013, 2015, 2018). They promote smooth forward movement of the center of gravity to keep the ankle joint in a higher degree of dorsiflexion (Ohata et al., 2011; Yamamoto et al., 2015). It has been reported that increasing the plantar flexion resistive moment significantly decreases the peak ankle plantar flexion and knee extension angle in the stance phase and increases the peak knee flexion moment and ankle joint power in the stance phase (Kobayashi et al., 2015). In addition, the changes depended on the level of plantar flexion resistance (Kobayashi et al., 2015, 2018). Therefore, AFO-PR may support heel rocker function by preventing foot drop and improving ankle dorsiflexion in the stance phase. However, they are not sufficient in supporting the forefoot rocker function during the toe-off phase with sufficient knee flexion.

In contrast, for patients with BKP, restriction of dorsiflexion is useful for restricting rapid motion of the tibia; however, it also restricts plantar flexion mobility in the loading response phase and causes insufficient ankle plantar flexion moment in the late stance phase (Mulroy et al., 2010). A previous study reported that articulated AFOs with plantar stop and free-dorsiflexion generated smooth dorsiflexion in the mid-stance phase; however, the ankle plantar flexion moment that they generated in the early and late stance phases was insufficient (Mulroy et al., 2010).

Recently, robotic control for disabled limbs has been developed. Compared with static controlled devices, robot-controlled devices can assist throughout the entire gait cycle according to the individual gait function and are expected to provide therapeutic effects by changing the degree of assistance. The last few years have seen the development of robotic AFOs designed to assist ankle movements, and studies have reported that they facilitated patients' gait and minimized occurrence of foot slap at initial contact by actively assisting ankle dorsiflexion in the swing phase (Yeung et al., 2017, 2018; Shi et al., 2019). However, most of these studies focused on assisting ankle dorsiflexion in the swing phase, with little consideration given to assisting ankle dorsiflexion in the stance phase.

An ankle joint walking assistive device “RE-Gait^®^” (Space Bio Laboratories, Japan) provides ankle plantar flexion and dorsiflexion in the stance and swing phases at the preferred timing, which is based on angular velocity. In previous studies, we focused on the device's assistance in forefoot rocker function and revealed its positive effects on gait function and spinal cord excitability (Tanaka et al., 2015; Nakagawa et al., 2020, 2022); however, the effects in the stance phase have not yet been examined. Therefore, we experimentally tested the effects of ankle control in the initial loading phase on the induction of an appropriate gait pattern using kinetic and kinematic analyses.

## Materials and methods

### Participants

Fifteen healthy adults (10 males, 5 females, age: 24.4 ± 4.4 years, height: 170.1 ± 9.6 cm, weight: 63.1 ± 12.2 kg) without lower limb orthopedic disease participated in this study. This study was approved in advance by the Ethics Committee of Hiroshima University in accordance with the Declaration of Helsinki, and written consent was obtained from all participants.

### Device

**Figure 1:**
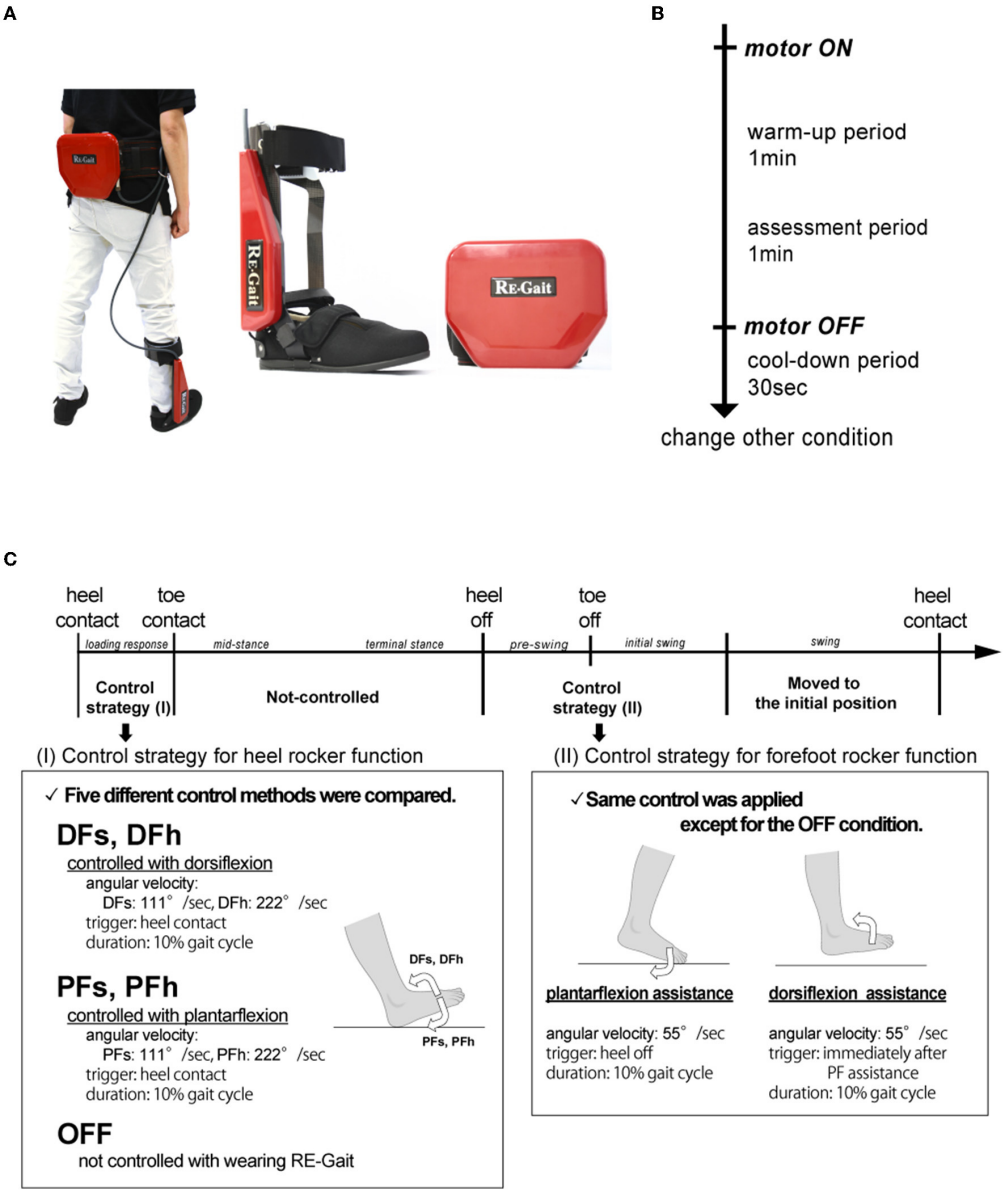
Experimental protocols. **(A)** RE-Gait. It consists of a controller (attached with the waist) and an ankle foot orthotic equipped with a motor and two sensors on the toe and heel. It is based on angular velocity control, and the controller generates the necessary torque using an engineering method. **(B)** Experimental protocol of each walking condition. **(C)** RE-Gait setting conditions. DFs, controlled with slow ankle dorsiflexion; DFh, controlled with high ankle dorsiflexion; PFs, controlled with slow ankle plantar flexion; PFh, controlled with high ankle plantar flexion; OFF, not controlled.

RE-Gait is a close-fitting assistive walking device that consists of a controller (attached to the waist belt, weight 900 g) and an ankle foot orthotic equipped with a motor and two sensors on the toe and heel (weight 1 kg) (Figure 1A). Only the weight of the orthotics corresponds to the mechanical structure attached to the user's ankle. The range of motion of the ankle joint was 50° in dorsiflexion and 40° in plantar flexion. If the control device is powered off, the resistance to the ankle is converted by the regeneration energy of the worm gear and motor. The device decodes the gait cycle using two pressure sensors located at the toe and heel, which control the angular velocity in each gait cycle at the preferred time, and the controller generates the necessary torque using an engineering method.

### Setup protocols

Participants were asked to walk on a treadmill (medical treadmill MAT-7000, Fukuda Denshi Co. Ltd., Tokyo, Japan) at a comfortable speed (2.0–2.8 km/h) under five conditions, wearing RE-Gait on their right leg. The comfortable speed for each participant was determined by measurements before the experiment.

Walking was performed for 2 min, with the first 1 min serving as a warm-up period, and the last 1 min as the assessed period. Furthermore, the participants walked for another 30 s with the device attached but not controlled by the motor to avoid the aftereffects of each walking condition (Figure 1B).

Five conditions were set depending on the initial control method from the heel contact to toe contact period (Figure 1C). The settings were controlled by angular velocity as follows: slow dorsiflexion robotically controlled to 111°/s (DFs); high dorsiflexion angular velocity controlled to 222°/s (DFh); slow plantar flexion controlled to 111°/s (PFs); high plantar flexion to 222°/s (PFh); and not controlled through the gait cycle (OFF). The duration of the assistance was set at 10% of each gait cycle period, and each condition was performed in a random order.

Subsequently, the control between the mid-stance and swing phase was set to be constant except for the OFF condition as follows: plantar flexion assistance (55°/s) for 10% of the gait cycle period after the heel off, and dorsiflexion assistance (55°/s) for 10% of the gait cycle period were applied immediately after the plantar flexion assistance, based on previous studies (Tanaka et al., 2015; Nakagawa et al., 2020, 2022).

### Kinematic assessment

We recorded their walking from the sagittal view with a high-speed video camera (iPhone Xs, frame rate: 120 fps) and assessed the joint angles of the knee and ankle, trailing limb angle (TLA), and minimum toe clearance (MTC) using markers attached to body feature points at the greater trochanter, lateral epicondyle of the knee, lateral malleolus, fifth metatarsal head of the right leg, and heel on the right leg. The marker of the lateral malleolus was placed perpendicular to the lateral malleolus on the device.

The joint angle was calculated by capturing the video and tracking the coordinates of the markers of each landmark using MATLAB R2020a Image Processing Toolbox and Computer Vision System Toolbox (MathWorks, Natick, USA). If the marker was not detected, linear interpolation was applied.

The knee joint angle was calculated as the angle between the thigh vector joining the greater trochanter with the lateral epicondyle and the lower leg vector joining the lateral epicondyle with the lateral malleolus. The ankle joint angle was calculated as the angle between the lower leg vector and the foot vector with the fifth metatarsal head and the heel. Changes in each gait cycle were analyzed by identifying two consecutive heel contacts. To analyze the angular changes, five periods were identified based on the toe and heel foot pressures: heel-contact, toe-contact, middle-stance, heel-off and toe-off. The middle-stance was defined as the intermediate period between toe-contact and heel-off. The gait cycle was divided into five phases according to the following five periods: loading response, mid-stance, terminal stance, pre-swing, and swing phases. Angular changes were calculated from peak-to-peak values in each phase.

The TLA was defined as the peak angle between the vertical axis and the vector joining the greater trochanter with the fifth metatarsal head (Hsiao et al., 2015a). The MTC was calculated by measuring the height from the parallel lines on the floor to the marker placed on the fifth metatarsal head and determining the minimum value in the swing phase.

### EMG assessment

EMG was also recorded using pre-amplified bipolar surface electrodes (FAD-SEMG1: 4Assist Inc., Tokyo, Japan), and the electrodes were placed on the tibialis anterior (TA), soleus (SOL), medial head of the gastrocnemius (MG), rectus femoris (RF), vastus medialis (VM), and biceps femoris (BF) of the right leg. After proper cleaning of the skin, EMG electrodes were placed over the belly of each muscle. The EMG signals were amplified ( × 100) using an EMG amplifier system (FAD-ABOX8, 4Assist Inc.) and digitized at 1000 Hz using Power Lab system (PowerLab 8/35, AD Instruments, Dunedin, New Zealand).

The EMG data were band-pass filtered at 10–200 Hz and rectified for offline analysis using LabChart v.8.1.12 (AD Instruments). They were then time-normalized and rectified into 100 data points for each gait cycle. The EMG amplitudes were normalized by the average amplitude of each muscle over the gait cycle for comparison of each condition (Kitatani et al., 2016) and were divided into five phases.

### Statistical analysis

The angular changes and, TLA, MTC, and EMG amplitudes in each phase were compared under the five different conditions (DFs, DFh, PFs, PFh, and OFF) using a one-way repeated measures analysis of variance. Before the analysis, we confirmed that the data were normality distributed using the Mauchly's sphericity test. If the significant differences were appeared, paired *t*-tests with Bonferroni's correction were carried out as *post-hoc* tests. Significance was set at *p* < 0.05 for each analysis. All statistical analyses were carried out using JMP software (version 15, IBM, New York, USA).

## Results

**Figure 2:**
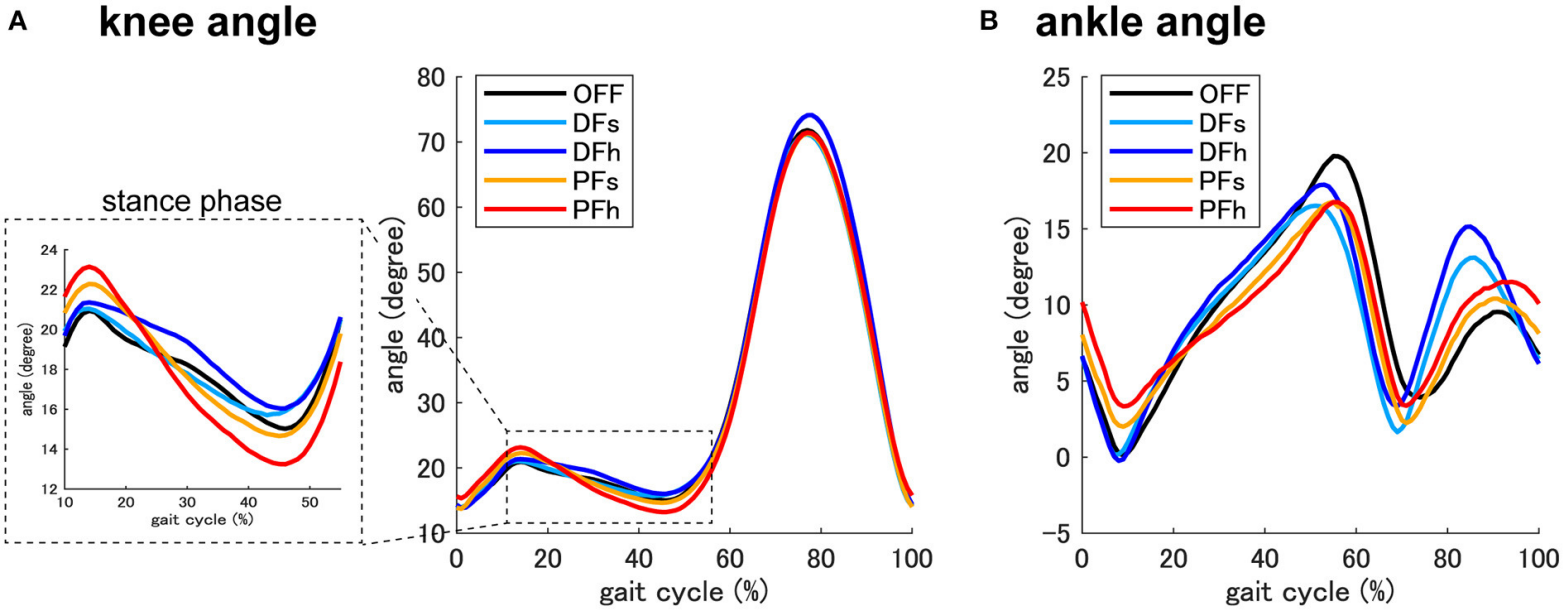
Knee and ankle joint angle. The averaged joint angle of the knee **(A)** and ankle **(B)** throughout the gait cycle (from the heel strike to the next heel strike of the right leg) in each condition. The dotted square shows a magnified view of the joint angle of the knee. DFs, controlled with slow ankle dorsiflexion; DFh, controlled with high ankle dorsiflexion; PFs, controlled with slow ankle plantar flexion; PFh, controlled with high ankle plantar flexion; OFF, not controlled.

The average changes in the knee and ankle joint angles throughout the gait cycle are shown in Figure 2. The changes in angles in each phase are listed in Table 1. Compared with the OFF condition (3.41 ± 2.72°), the PF condition enhanced the knee extension angle in the mid-stance phase (PFs: 5.09 ± 2.50°, PFh: 6.97 ± 4.35°), whereas the angle under the DFh condition was low (2.32 ± 2.45°). One-way repeated measures analysis of variance revealed a significant difference in the knee extension angle (F_(4, 56)_ = 12.19, *P* < 0.01). Moreover, the *post-hoc* test revealed that it was significantly increased under the PFh condition compared with that under the OFF condition.

**Table 1:** Joint movement in each phase.

	**OFF**	**DFs**	**DFh**	**PFs**	**PFh**	
**Knee joint Angle (degree)**						
Flexion in loading response	8.37 ± 4.32	7.81 ± 4.18	8.10 ± 4.18	9.66 ± 5.42	9.20 ± 5.81	F_4, 56_ = 1.74, *p* = 0.16
Extension in mid-stance	3.41 ± 2.72	3.60 ± 2.52	2.32 ± 2.45	5.09 ± 2.50	6.97 ± 4.35**	F_4, 56_ = 12.19, *p* < 0.01
Extension in terminal stance	3.42 ± 3.38	2.51 ± 2.49	3.72 ± 1.92	3.62 ± 3.24	4.48 ± 5.10	F_4, 56_ = 1.59, *p* = 0.19
Flexion in pre-swing	38.16 ± 5.17	36.96 ± 4.23	37.16 ± 6.40	36.75 ± 7.31	37.91 ± 5.85	F_4, 56_ = 0.40, *p* = 0.81
Flexion in-swing	19.25 ± 6.07	19.16 ± 5.91	21.65 ± 7.39	20.76 ± 6.85	21.58 ± 8.53	F_4, 56_ = 1.52, *p* = 0.20
**Ankle joint Angle (degree)**						
Plantarflexion in loading response	7.14 ± 3.77	6.93 ± 3.18	7.42 ± 3.68	7.02 ± 3.24	7.92 ± 3.45	F_4, 56_ = 0.54, *p* = 0.70
Dorsiflexion in mid-stance	10.36 ± 2.31	10.54 ± 2.82	11.66 ± 2.50	7.79 ± 3.54**	6.19 ± 3.90**	F_4, 56_ =18.44, *p* < 0.01
Dorsiflexion in terminal stance	10.46 ± 3.67	7.12 ± 2.21**	7.70 ± 2.79**	8.32 ± 2.31*	8.95 ± 3.14	F_4, 56_ = 7.69, *p* < 0.01
Plantarflexion in pre-swing	17.17 ± 3.36	16.21 ± 5.91	16.34 ± 6.11	15.31 ± 5.03	14.65 ± 3.89	F_4, 56_ = 1.63, *p* = 0.18
Dorsiflexion in-swing	7.21 ± 3.42	13.02 ± 4.76*	14.15 ± 6.41*	9.21 ± 5.00	9.07 ± 3.86	F_4, 56_ = 7.64, *p* < 0.01
**TLA in terminal stance (degree)**	16.99 ± 3.42	18.07*± 3.40	18.04*± 3.61	17.72 ± 3.66	17.98*± 4.02	F_4, 56_ = 3.64, *p* < 0.05
**MTC in swing (cm)**	6.16 ± 0.83	6.44 ± 1.28	7.04 ± 1.43**	6.32 ± 0.97	6.68 ± 1.11	F_4, 56_ = 6.06, *p* < 0.01

The angular data were calculated from the peak-to-peak values in each phase. The data were averaged value ± SD (standard deviation). The single asterisk (*) indicates a significant level of p < 0.05, and the double asterisk (**) indicates a significant level of p < 0.01 versus the OFF condition by the *post-hoc* tests.

OFF, not controlled; DFs, controlled with slow ankle dorsiflexion; DFh, controlled with high ankle dorsiflexion; PFs, controlled with slow ankle plantar flexion; PFh, controlled with high ankle plantar flexion; TLA, trailing limb angle; MTC, minimum toe clearance.

The ankle dorsiflexion angle also differed among the conditions; the timing of dorsiflexion was earlier under the DF conditions, and later under the PF conditions. Statistical analysis revealed significant differences in the dorsiflexion angle in the mid-stance phase (F_(4, 56)_ = 18.44, *P* < 0.01), terminal stance phase (F_(4, 56)_ = 7.69, *P* < 0.01), and swing phase (F_(4, 56)_ = 7.64, *P* < 0.01). *Post-hoc* tests showed that the dorsiflexion angle in the mid-stance phase was significantly lower under the PFs (7.79 ± 3.54°) and PFh (6.19 ± 3.90°) conditions than under the OFF (10.36 ± 2.31°) condition, and that in the terminal stance phase was significantly smaller under the DFs (7.12 ± 2.21°), DFh (7.70 ± 2.79°), and PFs (8.32 ± 2.31°) conditions than under the OFF condition. During the swing phase, the dorsiflexion angle in the DFh (14.15 ± 6.41°) condition was significantly higher than the OFF (7.21 ± 3.42°) condition.

The averaged results of the TLA and MTC under each condition are also shown in Table 1. Although the same assistance was applied between the mid-stance and initial swing phases, there were differences in toe trajectory under each condition. There were significant differences in the TLA and MTC under each condition (TLA: F_(4, 56)_ = 3.64, *P* < 0.05, MTC: F_(4, 56)_ = 6.06, *P* < 0.01). *Post-hoc* tests revealed that the TLA was significantly lower under the OFF condition than under the DFs, DFh, and PFh conditions. The MTC under the DFh condition was significantly higher than that under the OFF condition.

**Figure 3:**
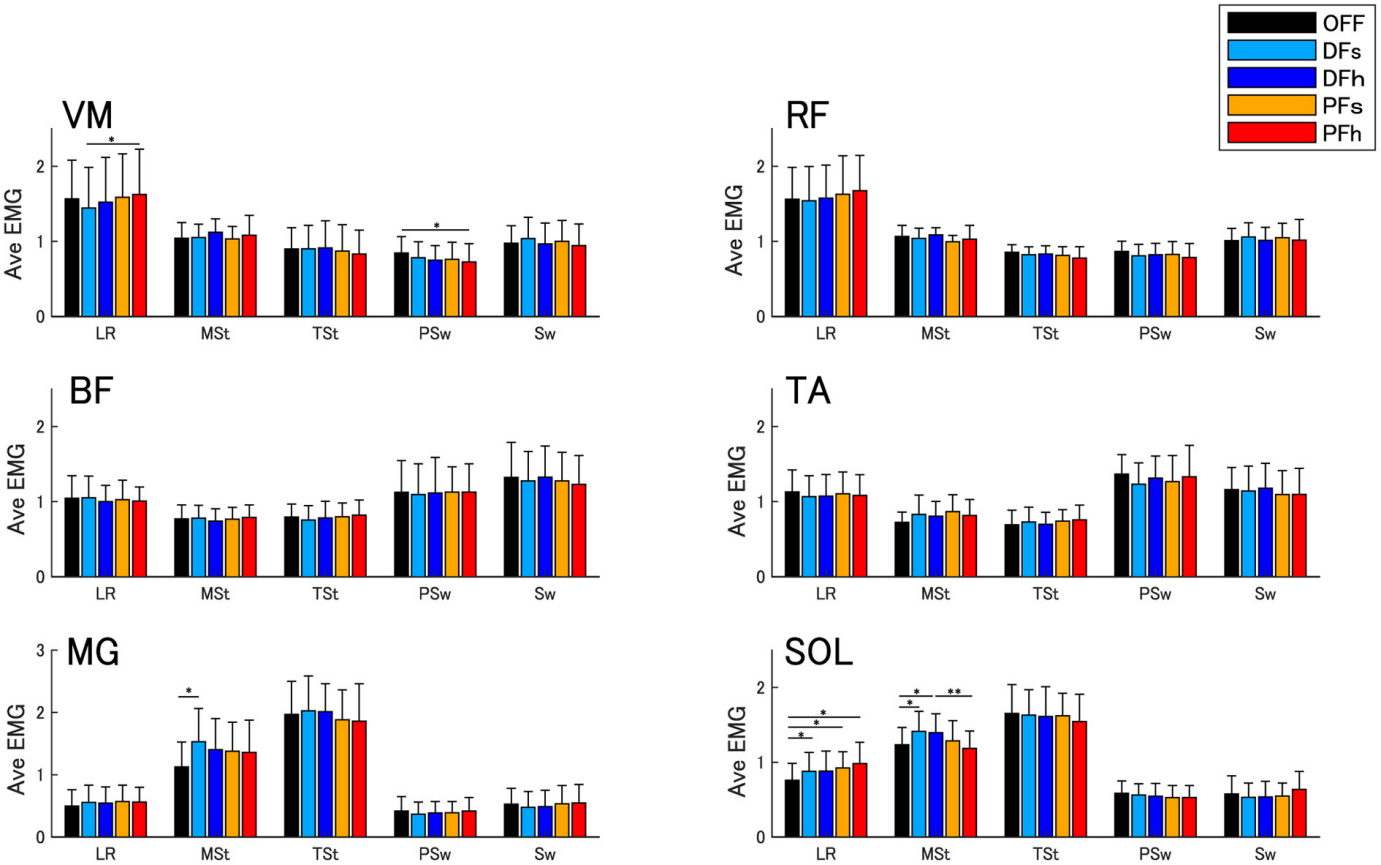
Averaged EMG activities. Error bars indicate standard deviations (SD). The single asterisk (*) indicates a significance level of *P* < 0.05, and the double asterisk (**) indicates a significance level of *P* < 0.01 with Bonferroni's correction. VM, vastus medialis; RF, rectus femoris; BF, biceps femoris; TA, tibialis anterior; MG, medial head of gastrocnemius; SOL, soleus; LR, loading response phase; MSt, mid-stance phase; TSt, terminal stance phase; PSw, pre-swing phase; Sw, swing phase.

Figure 3 shows the changes in the EMG activities in each phase. Some differences were found between the DF and PF conditions. The MG and SOL activation in the mid-stance phase was significantly higher under the DF conditions than under the OFF and PF conditions (MG: F_(4, 56)_ = 3.21, *P* < 0.05, SOL: F_(4, 56)_ = 5.41, *P* < 0.01). The VM activation under the PFh condition was significantly higher in the loading response phase (F_(4, 56)_ = 3.52, *P* < 0.05) and significantly lower in the pre-swing phase (F_(4, 56)_ = 3.38, *P* < 0.05).

## Discussion

In this study, the effects of ankle control using an ankle joint walking assistive device were assessed. The advantages of robotic ankle control are as follows: (1) ankle dorsiflexion and plantar flexion assistance in the initial loading phase control knee extension and ankle dorsiflexion in the stance phase, (2) assistance for the forefoot rocker function enhances TLA, and (3) obtaining smooth heel rocker function may have a positive effect on the lifting of the limb in the swing phase.

To improve the weight shift, appropriate rocker functions are needed (Czerniecki, 1988). The heel rocker function plays an important role in absorbing impact and generating an anterior propulsive force with efferent contraction of the TA and quadriceps femoris muscles. A previous study demonstrated that an increase in gait speed was correlated with an increase in braking forces during the initial loading phase (Nolan and Yarossi, 2011).

The present study showed that ankle assistance in the initial loading phase controlled the weight-bearing of the limb in the stance phase. In the stance phase, the knee extension angle was significantly increased under the PF condition, whereas obvious knee extension was not found under the DF conditions. The timing of the ankle dorsiflexion angle in the stance phase was earlier under the DF conditions but delayed under the PF conditions. Although no significant differences were found between the fast and slow angular velocity conditions, the changes tended to depend on the angular velocity intensity. These results indicate that DF settings in the initial loading phase may inhibit rapid strikes on the ground and promote moving tibial rotation, whereas PF settings may restrict rotation. Efficient gait was considered to be obtained by an inverted pendulum with proper ankle rocker function in the stance phase (Kuo, 2007), and the limb loading force in the initial loading phase was positively correlated with the propulsive force in the late stance phase (Hsiao et al., 2017). Hence, we hypothesized that ankle assistance in the initial loading phase would control tibial rotation in the early and late stance phases. These findings were supported by the EMG results. The ankle plantar flexors were considered to provide vertical and progressive support to the trunk throughout the stance phase. They accelerated the trunk vertically with decelerating forward trunk progression in the early and mid-stance phases (Neptune et al., 2001). Under the DF settings, significant EMG enhancement of MG and SOL activities was observed in the mid-stance phase, which suggests that they worked to control the forward progression of the tibia and produce potential energy in the early stance phase.

Post-stroke hemiplegic patients have difficulties in bearing and transferring weight onto the paretic limb from the initial loading to the terminal stance phase. The anterior propulsive force of the paretic limb was related to walking speed, hemiparetic severity, and step length asymmetry (Bowden et al., 2006; Balasubramanian et al., 2007; Hsiao et al., 2016b). Many previous studies have investigated ankle control in bearing and transferring weight using articulated AFOs. AFO-PR assisted the heel rocker function and reduced excessive gastrocnemius EMG activities in the loading response phase in stroke patients (Ohata et al., 2011); thus, AFO-PR achieved sufficient plantar flexion of the ankle by proper plantar flexion resistance torque during the loading response phase (Yokoyama et al., 2005). AFO-PR enhances walking ability (Yamamoto et al., 2015) and has been widely used clinically; however, it cannot adjust the amount of assistance and cannot support the patient during the entire gait cycle. However, AFOs with robotic assistance in the initial loading phase may be a novel approach for assisting with gait disturbance. The DF settings that produce smooth tibial rotation may be suitable for patients with SKP and ETP, while the PF settings are suitable for patients with BKP. In addition, the amount of assistance could be adjusted according to the improvement in the patient's gait disturbance.

Stroke patients have difficulty lifting the limb adequately, which often leads to toe-dragging, lower walking speed, shortened step length, and a high risk of tripping (Alam et al., 2014; Nagano et al., 2020). Inadequate push-off in the late stance phase causes lower toe clearance and knee flexion angle in the swing phase (Anderson et al., 2004; Campanini et al., 2013). However, few studies have reported the effects of providing ankle joint plantar flexion assistance with static AFOs (Sekiguchi et al., 2020). The present study provided adequate plantar flexion assistance in the pre-swing phase and dorsiflexion assistance in the initial swing phase except under the OFF condition. By inducing the plantar flexion torque, TLA was significantly enhanced under the robotic-assisted condition (DFs, DFh, PFh) compared with under the OFF condition, which is in line with our previous study that demonstrated TLA enhancement during and after RE-Gait intervention (Nakagawa et al., 2022). Plantar flexion assistance may improve forefoot rocker function, which contributes to the potential energy for lifting the heel and producing propulsion force. Previous studies have reported that TLA mainly contributes to an increase in propulsion force (Hsiao et al., 2015b, 2016a).

MTC occurs in the mid-swing phase at the point where the forward velocity of the foot is maximum (Begg et al., 2019) and is a predictor of tripping risk (Begg et al., 2007). Interestingly, MTC was significantly enhanced under the DFh condition compared with under the OFF condition, and the ankle joint angle in the swing phase was significantly increased under the DF condition. Although the same assistance was applied from the mid-stance to the swing phase, the effects of ankle lifting in the swing phase differed between DF and PF conditions. These findings suggest that providing appropriate assistance for obtaining a smooth weight shift with DF settings had a positive influence on lifting the limb in the swing phase. Efficient propulsive force and ankle plantar flexion torque lead to adequate hip extension and ankle plantar flexion in the pre-swing phase, which improves the push-off function and adequate toe clearance.

The present study investigated healthy adults; thus, further studies are needed to clarify the clinical application, as post-stroke hemiplegic patients present with various gait strategies. It is necessary to conduct research to verify the effects during use and the improvement effects before and after use in hemiplegic patients. Our findings in healthy adults suggest that robotic control of the ankle joint in the initial stance phase is a novel method for applying a proper walking pattern for neuromotor recovery.

## Conclusion

We investigated the effects of ankle control in healthy adults, using an ankle joint walking assistive device. We demonstrated that robotic ankle control in the initial stance phase may control movement throughout the gait cycle. Robotic ankle control may provide therapeutic effects by changing the degree of assistance according to the gait improvement.

## Data availability statement

The raw data supporting the conclusions of this article will be made available by the authors, without undue reservation.

## Ethics statement

The studies involving human participants were reviewed and approved by Ethics Committee of Hiroshima University. The participants provided their written informed consent to participate in this study.

## Author contributions

KN participated in the conceptualization, project administration, methodology, investigation, formal analysis, data curation, validation, supervision, visualization, and writing of the original draft. KH participated in the conceptualization, methodology, investigation, and writing-review and editing. AI participated in the investigation, formal analysis, and data curation. NK participated in the investigation and formal analysis. ET and LY participated in the supervision and writing-review and editing. All authors contributed to the article and approved the submitted version.

## Funding

This work was supported by a grant from the Japan Society for the Promotion of Science (22K11446), and funded by Space Bio Laboratories Co., Ltd. Part of this work was supported by the Center for Integrated Medical Research, at Hiroshima University Hospital.

## Conflict of interest

The authors declare that this study received funding from Space Bio-Laboratories Co., Ltd. The funder had the following involvement in the study: Provision of equipment. The funder was not involved in the study design, collection, analysis, interpretation of data, the writing of this article or the decision to submit it for publication. Author LY is employed by Space Bio-Laboratories Co., Ltd. The remaining authors declare that the research was conducted in the absence of any commercial or financial relationships that could be construed as a potential conflict of interest.

## Publisher's note

All claims expressed in this article are solely those of the authors and do not necessarily represent those of their affiliated organizations, or those of the publisher, the editors and the reviewers. Any product that may be evaluated in this article, or claim that may be made by its manufacturer, is not guaranteed or endorsed by the publisher.

## Abbreviations

AFO: ankle foot orthoses
AFO-PR: ankle foot orthoses with plantar flexion resistance
BKP: buckling knee pattern
BF: biceps femoris
DF: dorsiflexion
DFh: controlled with high ankle dorsiflexion
DFs: controlled with slow ankle dorsiflexion
EMG: electromyogram
ETP: extension thrust pattern
MG: medial head of gastrocnemius
MTC: minimum toe clearance
OFF: not controlled
PF: plantar flexion
PFh: controlled with high ankle plantar flexion
PFs: controlled with slow ankle plantar flexion
RF: rectus femoris
SKP: stiff knee pattern
SOL: soleus
TA: tibialis anterior
TLA: trailing limb angle
VM: vastus medialis.
